# Intellectual Structure and Emerging Trends of White Matter Hyperintensity Studies: A Bibliometric Analysis From 2012 to 2021

**DOI:** 10.3389/fnins.2022.866312

**Published:** 2022-04-11

**Authors:** Yanan Shi, Zehua Zhao, Huan Tang, Shijing Huang

**Affiliations:** ^1^Research and Development Center of Traditional Chinese Medicine, Guang’anmen Hospital, China Academy of Chinese Medical Sciences, Beijing, China; ^2^Graduate School, Beijing University of Chinese Medicine, Beijing, China

**Keywords:** white matter hyperintensities, bibliometrics, CiteSpace, VOSviewer, co-citation analysis, intellectual structure, hotspots, trends

## Abstract

White matter hyperintensities (WMHs), which have a significant effect on human health, have received increasing attention since their number of publications has increased in the past 10 years. We aimed to explore the intellectual structure, hotspots, and emerging trends of publications on WMHs using bibliometric analysis from 2012 to 2021. Publications on WMHs from 2012 to 2021 were retrieved from the Web of Science Core Collection. CiteSpace 5.8.R3, VOSviewer 1.6.17, and an online bibliometric analysis platform (Bibliometric. com) were used to quantitatively analyze the trends of publications from multiple perspectives. A total of 29,707 publications on WMHs were obtained, and the number of annual publications generally increased from 2012 to 2021. *Neurology* had the most publications on WMHs. The top country and institution were the United States and Harvard University, respectively. Massimo Filippi and Stephen M. Smith were the most productive and co-cited authors, respectively. Thematic concentrations primarily included cerebral small vessel disease, diffusion magnetic resonance imaging (dMRI), schizophrenia, Alzheimer’s disease, multiple sclerosis, microglia, and oligodendrocyte. The hotspots were clustered into five groups: white matter and diffusion tensor imaging, inflammation and demyelination, small vessel disease and cognitive impairment, MRI and multiple sclerosis, and Alzheimer’s disease. Emerging trends mainly include deep learning, machine learning, perivascular space, convolutional neural network, neurovascular unit, and neurite orientation dispersion and density imaging. This study presents an overview of publications on WMHs and provides insights into the intellectual structure of WMH studies. Our study provides information to help researchers and clinicians quickly and comprehensively understand the hotspots and emerging trends within WMH studies as well as providing direction for future basic and clinical studies on WMHs.

## Introduction

White matter hyperintensities (WMHs) are the neuroimaging features of cerebral small vessel disease (CSVD) and are commonly observed on brain magnetic resonance imaging (MRI) in older people (Wardlaw et al., 2015; Chen et al., 2019). The prevalence of WMHs is between 39% and 96% in the general population and increases with age (Prins and Scheltens, 2015). WMHs are related to cognitive impairment, stroke, depression, gait impairment, and death and influence many diseases (Wardlaw et al., 2015; Liu et al., 2017; de Havenon et al., 2019). Thus, the treatment of WMHs is of vital importance (Li Q. et al., 2018). WMHs have received significant attention, and relevant articles have been published. However, the pathogenesis of WMHs remains unclear, and no effective therapeutic measures are available (Li Q. et al., 2018).

There are a large number of publications related to WMHs. However, the previous publications were mainly focused on a single perspective of WMHs, lacking comprehensive bibliometric analysis on WMHs. Therefore, it is necessary to conduct a bibliometric study on WMHs.

Bibliometric analysis, also known as systematic scientometric review, is a type of systematic reviews that is performed with science mapping tools, including CiteSpace and VOSviewer software (Chen and Song, 2019). Using these tools, bibliometric analysis can quantitively analyze the publications, visualize the intellectual structure, measure the impact of publications, and identify emerging trends (Chen et al., 2012; Ellegaard and Wallin, 2015; Thompson and Walker, 2015; Agarwal et al., 2016). A network of diverse entities, including co-cited references, co-occurred keywords, and cooperative authors, is able to reflect the intellectual structure of a knowledge domain (Chen C.M. et al., 2014). Co-citation analysis is the basis of the visualization of intellectual structure within bibliometric analysis (Chen et al., 2012). Bibliometric analysis has some advantages over other knowledge synthesis approaches such as conventional expert-compiled reviews (Chen C.M. et al., 2014). Bibliometric analysis introduces quantitative analysis methods, which can obtain more objective results. With the help of bibliometric analysis tools, bibliometric analysis can input and analyze a much wider range of publications with higher analysis efficiency. However, conventional expert-compiled reviews are still irreplaceable as experts have profound and unique insights into a research field (Chen C.M. et al., 2014).

CiteSpace software, which contained information visualization methods, bibliometrics, and data mining algorithms, can visualize the co-citation network of scientific literature and identify trends and structure within a knowledge domain (Synnestvedt et al., 2005). CiteSpace integrates “time slice” networks to visualize the evolution of a knowledge domain over time. Scientific literature published in a 1-year time interval belongs to a “time slice” network (Chen C. et al., 2014). VOSviewer is another computer program for constructing and visualizing bibliometric networks (van Eck and Waltman, 2010). It is more advantageous in the visualization of large bibliometric maps. The network visualization, the overlay visualization, and the density visualization are the three types of visualizations in VOSviewer.

Web of Science (WoS), Scopus, and Google Scholar databases, which contain citation data, are commonly used in bibliometric studies (Kulkarni et al., 2009). WoS provides citation rates, which is extremely helpful, despite lacking citation tracking (Agarwal et al., 2016). Its depth of coverage is great, as numerous journals are covered from 1900 to present. Scopus is the largest abstract and citation database, while the depth of coverage is relatively poor. Although citation tracking is available, citation data are only available since 1996. Google Scholar is freely accessible, while it covers some sources that are non-scholarly (Agarwal et al., 2016).

Our study aimed to map the intellectual structure and illuminate the hotspots and trends of publications on WMHs from 2011 to 2021 using bibliometric methods. In this way, we can summarize the studies on WMHs in the past 10 years and provide some directions for future studies.

## Materials and Methods

### Data Sources and Search Strategies

The publications were retrieved from the Science Citation Index Expanded (SCI-EXPANDED) of the WoS Core Collection on 11 December, 2021. The search strategy was as follows: topic = (leukoaraiosis OR [white matter AND (hyperintensit* OR lesion* OR disease* OR change* OR abnormalit*)]) and time span = 2012–2021. Only original articles and reviews were included, and there were no other limitations. A total of 29,707 publications were obtained. We chose the past decade as the research time period for the study, as an analysis of studies published within the past decade enables researchers to identify the latest research trends. Only original articles and reviews were obtained, as these accounted for the vast majority of publications and contained all the items needed for bibliometric analysis.

### Analytical Method and Tools

Features of publications, such as journals and citations, were retrieved from WoS. The impact factor (IF), which reflects the influence of journals, was retrieved from the 2021 version of Journal Citation Reports (JCR).

The co-citation network is composed of references cited together by a set of articles and forms the intellectual base (Synnestvedt et al., 2005; Chen C. et al., 2014). Nodes, which represent references, are shown as citation “tree-rings” that represent the number of citations received in different years. Citation bursts, which means a surge of citations, are indicators of emerging trends (Chen C. et al., 2014). Citation bursts in specific time slices are represented by red rings in the network. Nodes with purple rings have higher betweenness centrality than other nodes, which means that their positions are critical in the network (Chen et al., 2012). The lines denote co-citation links.

Then nodes are clustered into groups representing different thematic concentrations. In the clustered network, the size of the nodes denotes the number of citations received (Chen C.M. et al., 2014). The smaller the serial number in the cluster label, the larger and more important the cluster is. The silhouette score of a cluster is close to 1, representing that the cluster has good homogeneity (Chen et al., 2012). The timeline view visualizes the changes of each cluster over time.

The keyword co-occurrence network, which can be visualized by VOSviewer, is able to indicate the hotspots (Chen et al., 2012). In the network, the nodes represent keywords, and the size of the nodes denotes the number of publications in which a keyword occurs. The lines represent co-occurrence links, and link strength represents the number of publications in which a keyword occurs. The nodes are divided into different clusters that are distinguished by color.

Cooperation analysis of countries and institutions, can identify the countries and institutions with great impact in the field of WMH studies and show the cooperation among them. The co-occurrence network of subject categories can reflect the hot disciplines and topics (Chen et al., 2012). Co-citation analysis of journals visualized by CiteSpace enables the identification of the influential journals on WMHs. Cooperation and co-citation analysis of authors are able to find out researchers making great contributions to WMH studies and visualize the cooperation between researchers.

The dataset of the 29,707 publications were input into CiteSpace. Different variants were merged in batches using a specific file. The time slicing was set as “from 2012 to 2021,” and years per slice was set to “1”. The link strength was set to “Cosine,” and the scope was set to “within slices”. The selection criteria used g-index in each slice, with the scale factor k value set to “25.” There was no network pruning. Co-citation analysis of references, journals, and authors, burst detection of keywords, and co-occurrence analysis of subject categories were performed with CiteSpace. In the timeline view of the clustered network of co-cited references, the top 12 clusters were displayed.

VOSviewer was used to visualize the cooperation analysis of countries, institutions, and authors and co-occurrence analysis of keywords. VOSviewer utilizes thesaurus files to merge different variants. In the cooperation analysis of countries, institutions, and authors, the maximum number of countries, institutions, or authors per document was set to “25”. The minimum number of documents of a country, institution, or an author was set to “5”, and the minimum number of citations of a country, institution, or an author was set to “0”. The top 50 countries, top 100 institutions, and top 100 authors were displayed in the networks.

An online bibliometric analysis platform^1^ and Microsoft Excel were used to map the publication distribution.

The number of publications by countries and institutions refers to the number of all articles published by authors from these countries and institutions in the dataset obtained from WoS. The citations of publications by countries and institutions were received not only from the field of WMHs, but also from other research fields in WoS. Therefore, the number of the citations reflects the impact of these publications on all fields, not only within the field of WMHs. In the co-citation analysis of references, the number of citations refers to the times a reference was co-cited with other references in the obtained dataset. With respect to the co-citation analysis of journals and authors, the number of citations refers to the times a reference including the specified journals or authors was co-cited with the other references in the obtained dataset. In the author cooperation analysis, article counts refer to the number of articles published by the authors in the obtained dataset. In the subject category co-occurrence analysis, frequency refers to the number of articles that belong to the subject categories in the obtained dataset. In the keyword co-occurrence analysis, frequency refers to the number of articles in which a given keyword occurred within the obtained dataset.

## Results

### Yearly Quantitative Distribution of Articles

**FIGURE 1:**
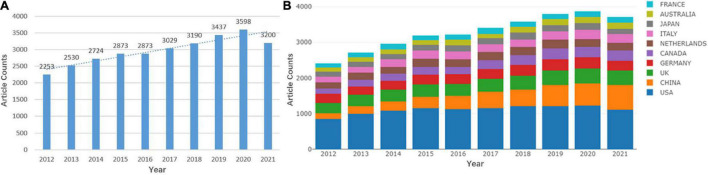
Quantitative distribution of publications on white matter hyperintensities (WMHs). **(A)** Annual distribution of publications on WMHs. **(B)** Annual distribution of publications on WMHs among the top 10 countries.

The annual number of publications can indicate the overall trend of WMH studies. From 2012 to 2021, a total of 29,707 original articles and reviews were included. The overall trend of the annual number of publications increased, from 2,253 in 2012 to 3,200 in 2021 (Figure 1A). Figure 1B shows that the United States has maintained the largest number of publications, whereas the publications of China has continued to grow rapidly in the past decade.

### Country and Institution Cooperation Analysis

Cooperation analysis of countries and institutions is used to explore countries and institutions with enormous influence and the collaboration between them.

**FIGURE 2:**
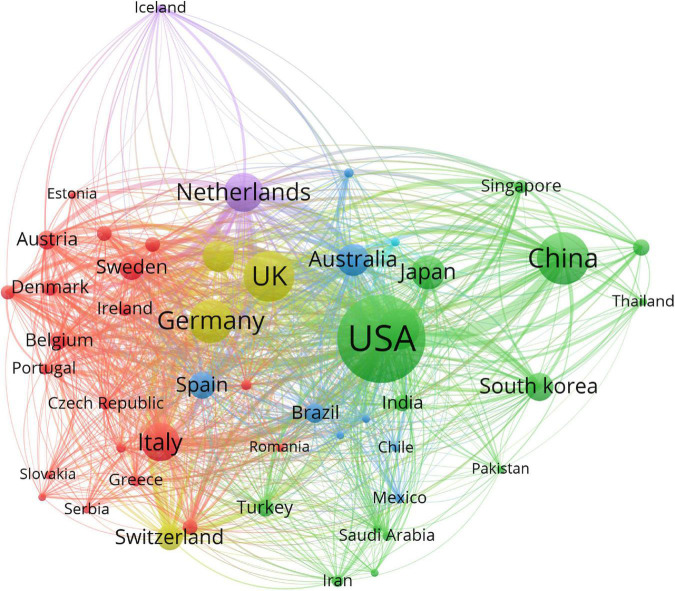
Cooperation network of countries and regions contributed to publications on white matter hyperintensities. The nodes represent the countries, and the lines represent the cooperation between countries. The size of the nodes depicts the number of publications. The color of the nodes distinguishes the cluster of the countries.

The 29,707 articles were distributed among 126 countries and regions. As shown in Table 1, the leading country in terms of the number of publications is the United States (11,225, 37.0%), followed by China (3,905, 13.1%), the United Kingdom (3,672, 12.4%), Germany (2,800, 9.4%), and Canada (2,386, 8.0%). These countries made great contributions to WMH studies. The United States had the maximum total citations (31,5421), followed by the United Kingdom (120,975) and Netherlands (76,885). In the cooperation network (Figure 2), the size of the nodes depicts the number of publications, and the link strength represents the closeness of the collaborations. Countries and regions with the top five link strength were the United States, the United Kingdom, Germany, Netherlands, and Canada. These countries had more cooperation than other countries.

**TABLE 1:** The top 10 countries and regions contributing to the publications on white matter hyperintensities.

Rank	Country/region	Article counts	Total number of citations	Average number of citations	Total link strength
1	United States	11,225	315,421	28.10	8469
2	China	3,905	53,136	13.61	2080
3	United Kingdom	3,672	120,975	32.95	5951
4	Germany	2,800	76,885	27.46	4331
5	Canada	2,386	62,704	26.28	3195
6	Netherlands	2,181	67,745	31.06	3505
7	Italy	2,156	50,441	23.40	2992
8	Japan	1,674	25,765	15.39	908
9	Australia	1,475	39,439	26.74	2284
10	France	1,455	40,599	27.90	2447

**FIGURE 3:**
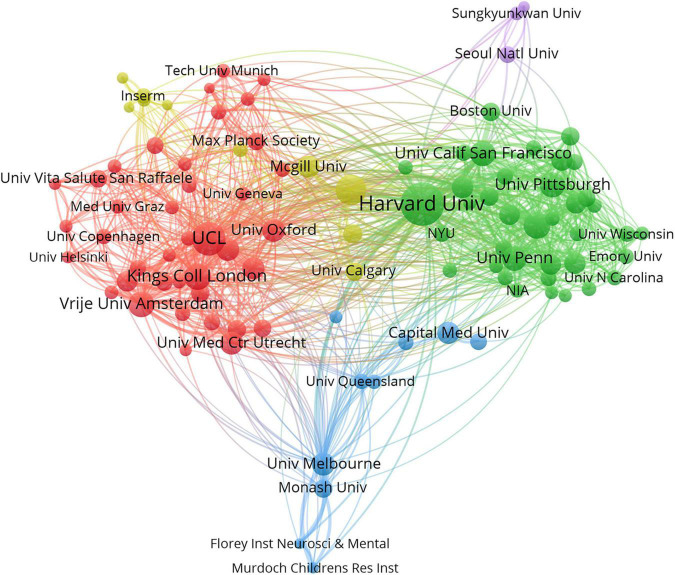
Cooperation network of institutions contributed to publications on white matter hyperintensities. The nodes represent the institutions, and the lines represent the cooperation between institutions. The size of the nodes denotes the number of publications. The color of the nodes distinguishes the cluster of the institutions.

A total of 16,677 institutions published articles on WMHs. As shown in Table 2, Harvard University has the maximum number of publications (1,525, 5.1%), followed by University College London (898, 3.0%), the University of Toronto (743, 2.5%), Johns Hopkins University (586, 2.0%), and King’s College London (563, 1.9%). Therefore, Harvard University, University College London, the University of Toronto, Johns Hopkins University, and King’s College London were the influential institutions in the field of WMH studies. Harvard University was the most cited institution (48,292), followed by University College London (28,722) and Vrije University Amsterdam (19,956). As shown in Figure 3, institutions with the top five link strength are Harvard University, University College London, University of California San Francisco, Vrije University Amsterdam, and King’s College London. These institutions had more cooperation than other institutions.

**TABLE 2:** The top 10 institutions contributing to publications on white matter hyperintensities.

Rank	Institutions	Article counts	Total number of citations	Average number of citations	Total link strength
1	Harvard University	1525	48,292	31.67	1993
2	University College London	898	28,722	31.98	1407
3	University of Toronto	743	16,511	22.22	767
4	Johns Hopkins University	586	15,421	26.32	703
5	King’s College London	563	19,824	35.21	804
6	Vrije University Amsterdam	533	19,956	37.44	866
7	University of California San Francisco	519	17,118	32.98	890
8	University of Pittsburgh	507	17,600	34.71	562
9	University of Pennsylvania	482	15,747	32.67	714
10	University of Cambridge	420	14,320	34.10	586

### Journal Co-citation Analysis

Co-citation analysis of journals aims to identify journals with great influence and illustrate the associations among journals. The top 20 journals are listed in Table 3. *Neurology* had the maximum number of citations (16,180 citations), with an IF of 9.910. *Neuroimage* was the second most cited journal (15,805 citations), with an IF of 6.556. *Brain*, which had an IF of 13.501, ranked third with a citation count of 14,385. They were the journals with great impact. *Neuroimage* had the largest centrality value (0.06), which demonstrated that *Neuroimage* had an important position in the network. According to the JCR 2021 standards, eight of the top 10 journals were categorized as the first quartile (Q1), and two of the top 10 journals belonged to the second quartile (Q2). The top 10 journals were the mainstream journals in the field.

**TABLE 3:** The top 10 co-cited journals on white matter hyperintensities.

Rank	Journal title	Total number of citations	Centrality	Impact factor(2021)	Quartile in category (2021)
1	*Neurology*	16,180	0.04	9.91	Q1
2	*Neuroimage*	15,805	0.06	6.556	Q1
3	*Brain*	14,385	0.03	13.501	Q1
4	*PLos ONE*	12,765	0.03	3.24	Q2
5	*Annals of Neurology*	11,397	0.01	10.422	Q1
6	*Journal of Neuroscience*	10,821	0.05	6.167	Q1
7	*Proceedings of the National Academy of Sciences of the United States of America*	9,776	0.03	11.205	Q1
8	*American Journal of Neuroradiology*	9,739	0.01	3.825	Q2
9	*Human Brain Mapping*	9,170	0.02	5.038	Q1
10	*Journal of Neurology Neurosurgery and Psychiatry*	9,129	0.01	10.154	Q1
					

### Author Cooperation and Co-citation Analysis

**FIGURE 4:**
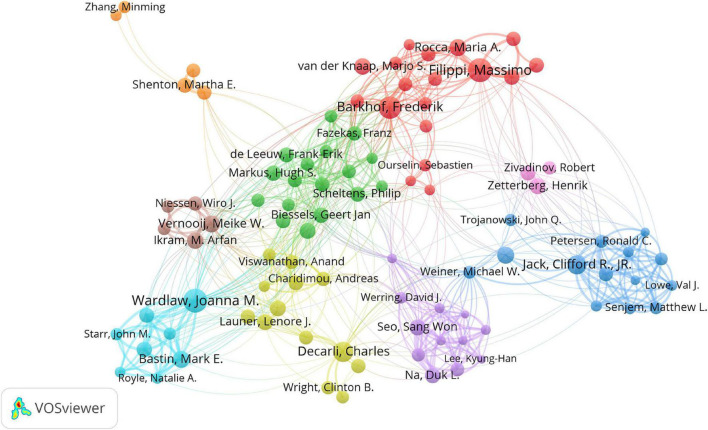
Cooperation network of authors contributed to publications on white matter hyperintensities. The nodes represent the authors, and the lines represent the cooperation between authors. The size of the nodes represents the number of publications. The color of the nodes distinguishes the cluster of the authors.

Cooperation and co-citation analysis of authors aim to identify researchers with great impact in the research field and promote the collaboration among researchers. The top 10 productive authors and co-cited authors are listed in Table 4. The cooperation of the top 100 productive authors is illustrated in Figure 4. Massimo Filippi, from Vita-Salute San Raffaele University in Italy, published the highest number of articles, followed by Joanna M. Wardlaw from University of Edinburgh in the United Kingdom and Frederik Barkhof from University College London in the United Kingdom and Amsterdam University Medical Centers in the Netherlands. They were the most productive authors. Smith S.M. (4,549 citations) was the most cited author, followed by Wardlaw J.M. (2,152 citations), Basser P.J. (2,101 citations), and Fazekas F. (2,054 citations). They had great influence in the field of WMH studies.

**TABLE 4:** The top 10 productive authors and co-cited authors in white matter hyperintensity studies.

Rank	Author	Article counts	Co-cited author	Total number of citations
1	Massimo Filippi	168	Smith SM	4,549
2	Joanna M. Wardlaw	165	Wardlaw JM	2,152
3	Frederik Barkhof	143	Basser PJ	2,101
4	Charles Decarli	116	Fazekas F	2,054
5	Clifford R. Jack Jr	108	Mori S	2.011
6	Mark E. Bastin	85	Jenkinson M	1,996
7	Meike W. Vernooij	85	Ashburner J	1,975
8	Maria A. Rocca	82	Fischl B	1,802
9	Paul M. Thompson	82	Pantoni L	1,720
10	Qiyong Gong	80	Song SK	1,573

### Subject Category Co-occurrence Analysis

Every periodical of WoS belongs to one or more subject categories. The co-occurrence analysis of subject categories can indicate the influential disciplines. Burst detection illustrated active study areas. Neurosciences and Neurology was the largest category with the highest number of publications, followed by Neurosciences and Clinical Neurology (Table 5). By the end of 2021, bursts of subject categories included “computer science, information system,” “telecommunications,” and “engineering, electrical and electronic,” etc., which indicated the emerging trends (Supplementary Figure 1). Therefore, computer science, information technology, and electronic technique are increasingly being applied in WMH studies.

**TABLE 5:** The top 10 subject categories.

Rank	Category	Freq
1	Neurosciences and Neurology	17,715
2	Neurosciences	11,370
3	Clinical Neurology	8,845
4	Radiology, Nuclear Medicine and Medical Imaging	3,885
5	Neuroimaging	3,229
6	Psychiatry	2,521
7	Science and Technology-Other Topics	1,567
8	Multidisciplinary Sciences	1,546
9	Geriatrics and Gerontology	1,323
10	Cardiovascular System and Cardiology	1,151

### Keyword Co-occurrence Cluster Analysis

Keyword co-occurrence cluster analysis is used to explore core keywords, identify the co-occurrence links, classify keywords into clusters, and shed light on the hotspots. Burst detection reflects emerging trends (Chen C.M. et al., 2014).

**FIGURE 5:**
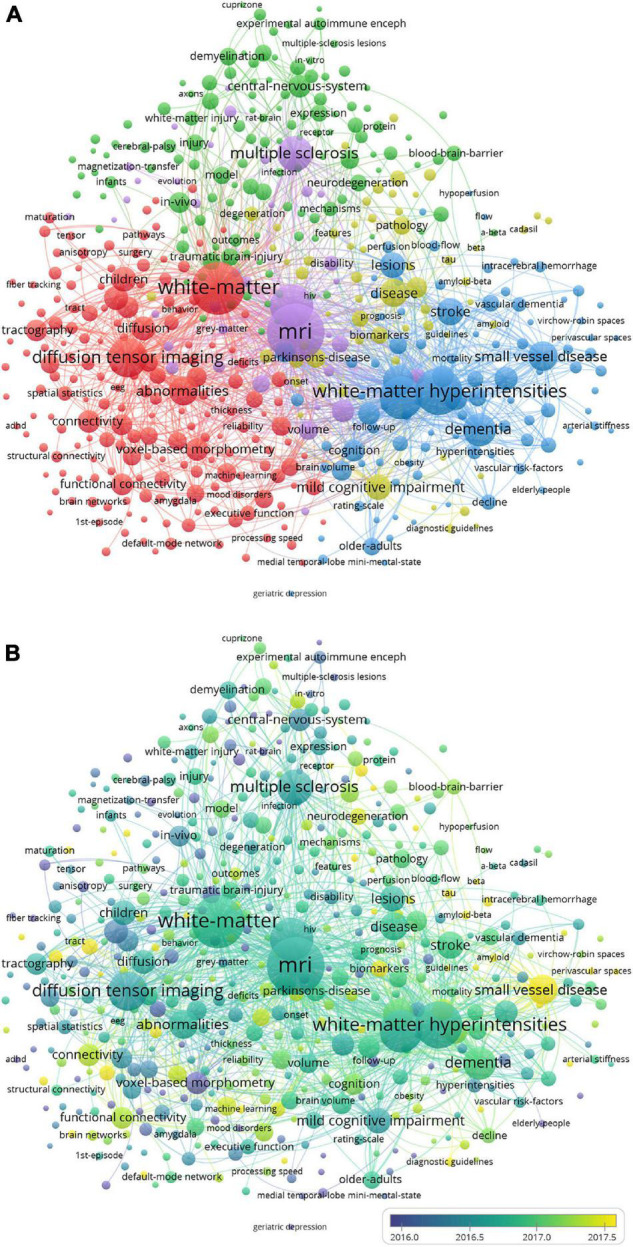
Clustered network of keyword co-occurrence. **(A)** Network visualization of keyword co-occurrence cluster analysis. **(B)** Overlay visualization of keyword co-occurrence cluster analysis. The keywords are depicted as the nodes, and the lines represent the co-occurrence links. The size of the nodes denotes the number of publications. In panel **(A)**, the color of the nodes distinguishes the cluster of the keywords. In panel **(B)**, the color of the nodes, which denotes the average publication year, changes from dark blue to yellow, representing the average publication year of the keyword from 2012 to 2021.

The network of keyword co-occurrence cluster analysis is shown in Figure 5A. The 7,855 keywords with a frequency over five were analyzed and divided into five clusters: white matter and diffusion tensor imaging (DTI) (in red), inflammation and demyelination (in green), small vessel disease (SVD) and cognitive impairment (in blue), MRI and multiple sclerosis (MS) (in yellow), and Alzheimer’s disease (AD) (in purple). They were the hotspots of the research field. The temporal distribution of the keywords co-occurrence cluster is shown in Figure 5B. The color of the nodes depicts the average publication year and changes from dark blue to yellow, representing the average publication year of the keywords from 2012 to 2021. SVD, machine learning (ML), connectivity, tract, and neurodegeneration, etc., which have approximate yellow colors, have recent average publication time. Therefore, these keywords have been the active topics in recent years.

**FIGURE 6:**
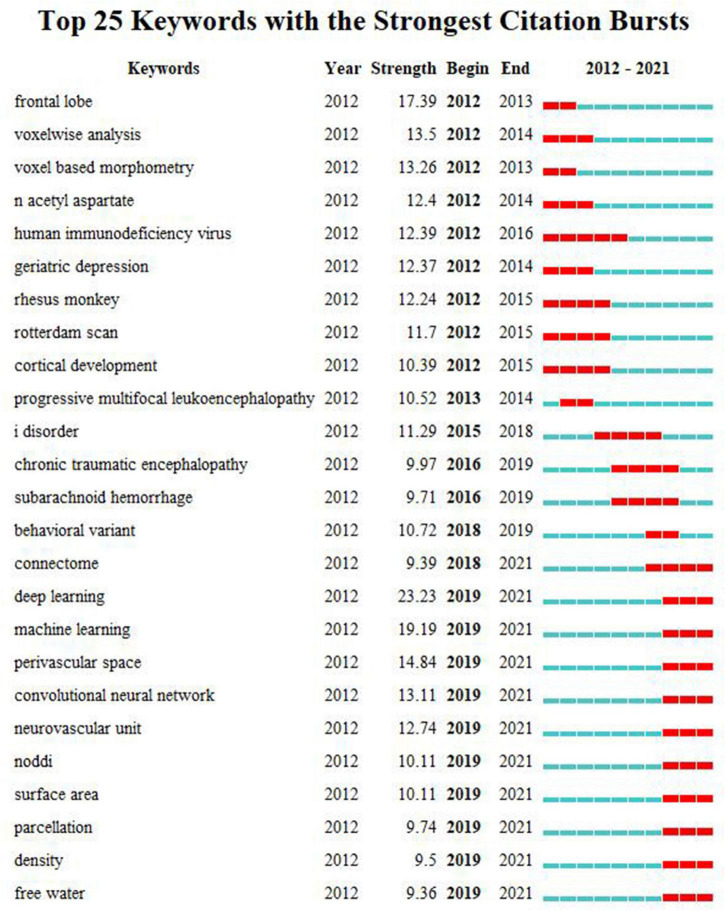
The top 25 keywords with the strongest citation bursts during 2012–2021. Each line denotes a keyword with its occurrence year, the citation burst strength, and the beginning and ending times of the citation burst. On the right side of the figure, each line, which is divided into 10 short lines, represents the time from 2012 to 2021. Each red line segment depicts a citation burst in the corresponding year.

The top 25 keywords with the strongest citation bursts were detected from 2012 to 2021. As shown in Figure 6, by the end of 2021, deep learning (DL) has the maximum burst strength, followed by ML, perivascular space (PVS), convolutional neural network (CNN), neurovascular unit (NVU), neurite orientation dispersion and density imaging (NODDI), surface area, parcellation, density, connectome, and free water. They were the emerging trends in the research of WMHs.

### Co-cited Reference Analysis

The co-citation analysis of references can identify frequently cited references and explore the relationships between references.

References cited by the 29,707 articles were analyzed and visualized. Table 6 displays the top 10 co-cited references. The article published by Wardlaw et al. in the *Lancet Neurology* had the maximum number of citations (658 citations), followed by the article published by Andersson and Sotiropoulos in the *Neuroimage* (410 citations) and the article published by Jenkinson et al. in the *Neuroimage* (358 citations) (Jenkinson et al., 2012; Wardlaw et al., 2013b; Andersson and Sotiropoulos, 2016). These references had important implications for WMH studies.

**TABLE 6:** The top 10 co-cited references.

Rank	Title	First author	Journal	Year	Total citations
1	Neuroimaging standards for research into small vessel disease and its contribution to ageing and neurodegeneration (Wardlaw et al., 2013b)	Joanna M. Wardlaw	*Lancet Neurol*	2013	658
2	An integrated approach to correction for off-resonance effects and subject movement in diffusion MR imaging (Andersson and Sotiropoulos, 2016)	Jesper L R. Andersson	*Neuroimage*	2016	410
3	FSL (Jenkinson et al., 2012)	Mark Jenkinson	*Neuroimage*	2012	358
4	White matter integrity, fiber count, and other fallacies: the do’s and don’ts of diffusion MRI (Jones et al., 2013)	Derek K. Jones	*Neuroimage*	2013	357
5	Permutation inference for the general linear model (Winkler et al., 2014)	Anderson M. Winkler	*Neuroimage*	2014	304
6	Diagnostic criteria for multiple sclerosis: 2010 revisions to the McDonald criteria (Polman et al., 2011)	Chris H. Polman	*Annals of Neurology*	2011	297
7	Threshold-free cluster enhancement: addressing problems of smoothing, threshold dependence and localisation in cluster inference (Smith and Nichols, 2009)	Stephen M. Smith	*Neuroimage*	2009	283
8	The clinical importance of white matter hyperintensities on brain magnetic resonance imaging: systematic review and meta-analysis (Debette and Markus, 2010)	Stéphanie Debette	*BMJ*	2010	274
9	Mechanisms of sporadic cerebral small vessel disease: insights from neuroimaging (Wardlaw et al., 2013a)	Joanna M. Wardlaw	*Lancet Neurol*	2013	251
10	White matter hyperintensities, cognitive impairment and dementia: an update (Prins and Scheltens, 2015)	Niels D. Prins	*Nat Rev Neurol*	2015	246

### Co-cited Reference Cluster Analysis

The co-cited reference cluster analysis can devide references into clusters that represent different thematic concentrations, in order to shed light on the intellectual structure.

**FIGURE 7:**
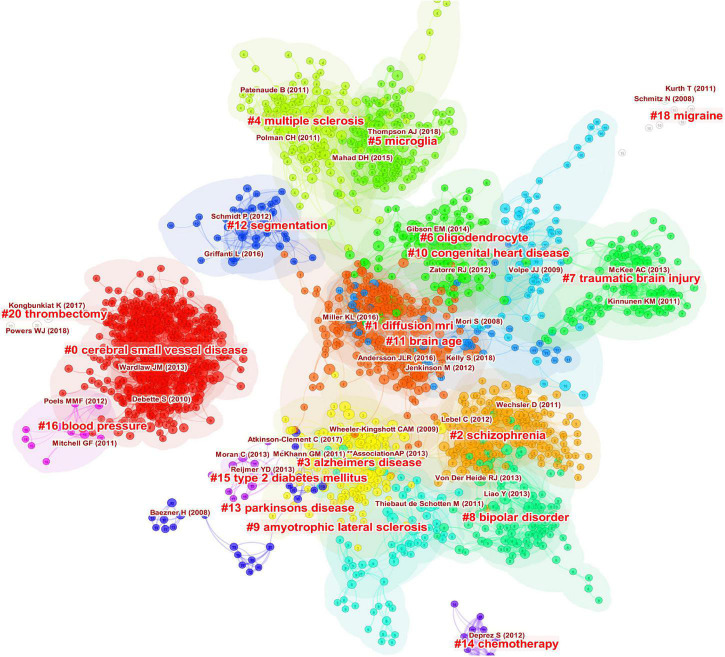
Clustered network of co-cited references of the publications on white matter hyperintensities. The nodes represent the references, and the lines represent the co-citation links between the references. The size of the nodes denotes the number of citations. The color of the nodes distinguishes the cluster of the references. The labels in bright red are the names of the clusters, and the labels in dark red represent the important articles highly cited of each cluster.

As shown in Figure 7, the references are clustered and analyzed, and the seven largest clusters include CSVD, diffusion MRI (dMRI), schizophrenia, AD, MS, microglia, and oligodendrocyte. Every cluster denotes a thematic concentration of intellectual structure. The network contains 1955 nodes and 9899 links. The weighted mean silhouette score is 0.909, which means that the clusters had good homogeneity.

**FIGURE 8:**
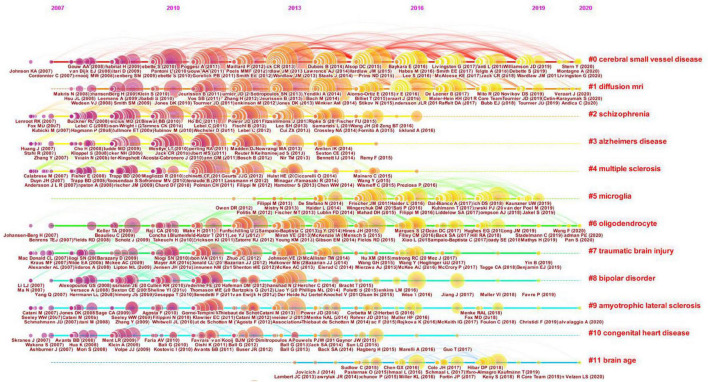
Timeline view of the clustered network of co-cited references. Every cluster is shown on a horizontal line with a cluster label on the right. The time is displayed at the top of the figure, which can be referred to judge the publication time of the references. The references are shown as citation rings which reflect their citations, and the reference labels are shown under the citation rings. The lines depict the co-citation links. The color of the lines represents different clusters.

The timeline view illustrates the distributions and changes of nodes in each cluster over time, which are reflective of changes in research focus. The timeline view of the co-citation cluster analysis of references is shown in Figure 8. Every cluster is shown on a horizontal line with a cluster label on the right. The time is at the top, which can be used to judge the publication time. The references are depicted as citation rings that reflect their citations, and the reference labels are shown under the citation rings. The lines represent the co-citation links between nodes. The color of the lines distinguishes different clusters. The timeline view can reflect the trends of the research field over time, highlighting emerging and pivotal study directions. As shown in Figure 8, cluster #0 CSVD and cluster #1 dMRI are the largest clusters. These two clusters appeared early and persisted. Cluster #5 microglia and #cluster 11 brain age occurred late. These results suggest that CSVD and dMRI has been the major focuses of researchers in this field during the past decade. However, microglia and brain age have attracted researchers’ attention in recent years.

## Discussion

This study quantitatively analyzed the publications on WMHs from 2012 to 2021 using the bibliometric analysis method. With the quantitative method, it provided an overview of WMH studies, characterized the intellectual structure, and identified the emerging trends. Other knowledge synthesis methodologies such as conventional reviews have also been used in the field of WMH studies (Prins and Scheltens, 2015). However, conventional reviews use subjective and qualitative analytical methods and mainly focus on one aspect of WMH studies. Thus, it is difficult to get a true overview of the research field. Therefore, bibliometric analysis may be a superior method for quantitatively analyzing publications.

### General Trends of Publications on White Matter Hyperintensities

The number of annual publications on WMHs in the past decade has been generally increasing, indicating that WMHs have received increasing attention. Therefore, the importance of WMH studies has been increasingly recognized.

*Neurology* had the maximum number of citations, followed by *Stroke* and *Annals of Neurology*, indicating that they were influential journals on WMHs. The top 10 cited journals were the mainstream journals in the field of neurology, which demonstrates that research on WMHs is a hot topic in neurology.

The United States, China, and the United Kingdom were the three countries that published the maximum number of articles on WMHs. The United States, the United Kingdom, and Germany were the leading countries in the cooperation network. Therefore, the United States and the United Kingdom were the most influential countries in the field of WMHs. China has the second largest number of publications on WMHs. However, China’s cooperation strength in the cooperation network was relatively low, which indicates that China should enhance its cooperation with other countries.

Harvard University had the most publications, followed by University College London and the University of Toronto. It’s obvious that Harvard University leads the way in WMH studies. Five of the top 10 countries that contributed to publications on WMHs were in the United States, which indicates that institutions in the United States contributes the most to publications on WMHs. Harvard University, University College London, University of California San Francisco, Vrije University Amsterdam, and King’s College London collaborate more than other institutions.

### Intellectual Structure of Publications on White Matter Hyperintensities

The intellectual structure is composed of multiple entities, including co-cited references and co-occurring keywords (Chen C.M. et al., 2014).

Massimo Filippi published the highest number of articles, followed by Joanna M. Wardlaw and Frederik Barkhof. Thus, they are the most prolific authors. Stephen M. Smith was the most cited author, followed by Joanna M. Wardlaw and Peter J. Basser. Therefore, their publications had a significant impact on WMH studies.

The subject category co-occurrence analysis demonstrated that computer science, information technology, and electronic technique have been increasingly introduced into WMH studies, acting as advanced tools in the study of WMHs.

The top 10 co-cited references had significant implications for WMH studies. The most cited reference, which was written by an international working group, proposed a series of detailed neuroimaging standards for CSVD; it removed a significant barrier and laid a solid foundation for the study and clinical practice of CSVD (Wardlaw et al., 2013b). The second top cited reference developed a method to estimate and correct for off-resonance induced distortions and subject movement in diffusion imaging, which has been widely used (Andersson and Sotiropoulos, 2016). The third top cited reference systematically introduced the development process and current situation of the FMRIB Software Library, which is widely used to analyze MRI brain imaging data (Jenkinson et al., 2012).

There were three other top cited references on neuroimaging. Jones et al. (2013) provided insights into the physics of diffusion-weighted MRI, appropriate approaches to obtain diffusion-weighted MRI data, method to interpret the results, and a list of the things that should be done or avoided. Winkler et al. (2014) proposed permutation inference for a general linear model to control false positives in imaging research scenarios. Smith and Nichols (2009) presented a threshold-free cluster enhancement method that was more sensitive and stable than cluster-based thresholding.

The other four top cited references are on white matter diseases. Polman et al. (2011) introduced the revision of the diagnostic criteria for multiple sclerosis, the McDonald criteria, in 2010, with the simplification of the criteria and the emphasis on the application in broader populations. Debette and Markus (2010) conducted a meta-analysis and found that the increased risk of stroke, dementia, and death can be estimated using WMHs. Wardlaw et al. (2013a) proposed the potential pathogenesis of SVD from the perspective of neuroimaging and presented hypotheses for the study of SVD. Prins and Scheltens (2015) suggested that WMHs were predictors of cognitive decline and could be a therapeutic target.

The keywords can be divided into five clusters: white matter and DTI, inflammation and demyelination, SVD and cognitive impairment, MRI and MS, and AD. These were the hotspots in WMH studies. The main clusters of co-cited references were CSVD, dMRI, schizophrenia, AD, MS, microglia, and oligodendrocyte, which were the thematic concentrations of the WMH publications. CSVD and dMRI have been the major thematic concentrations in the decade, while microglia and brain age have attracted researchers’ attention only in recent years. Hotspots and thematic concentrations were intrinsically linked.

Cerebral small vessel disease is a broad spectrum of diseases affecting the perforating arterioles, capillaries, and venules with heterogeneous etiologies. Recent small subcortical infarcts, lacunes, WMHs, PVSs, microbleeds, and brain atrophy are the neuroimaging biomarkers of CSVD (Wardlaw et al., 2013b). The clinical manifestations of CSVD are heterogeneous, containing cognitive decline, psychiatric disorders, gait dysfunction, and urinary incontinence (Cannistraro et al., 2019). The pathogenesis of CSVD needs to be further elucidated, and CSVD lacks specific prevention and treatment methods; currently, vascular risk factors are the targets for the prevention and treatment of CSVD (Li Q. et al., 2018; Cannistraro et al., 2019).

Diffusion imaging, which utilizes the water diffusion–driven displacement (Le Bihan, 2013), can differentiate individual tracts and assess both the microstructure and macrostructure of white matter. Diffusion imaging is a popular method despite its shortcomings, such as lack of specificity (Lebel et al., 2019). Diffusion weighted imaging, DTI, advanced multi-compartment diffusion models, tractography analysis, and connectivity analysis all belong to the category of diffusion imaging (Raja et al., 2019). DTI, which utilizes the diffusion tensor model to map the white matter fiber (Basser et al., 1994a,b), has a broader range of uses than any other diffusion methods despite some limitations (Jones and Cercignani, 2010; Tournier et al., 2011). Mean diffusivity and fractional anisotropy are the DTI scalars used to measure the properties of white matter. Advanced diffusion methods such as diffusion kurtosis imaging can characterize the white matter microstructure more specifically (Jensen et al., 2005; Xu et al., 2016).

Schizophrenia is a serious psychiatric disease with apparent clinical heterogeneity (Tan et al., 2020). Widespread white matter is a prominent neuroimaging feature of schizophrenia (Cetin-Karayumak et al., 2020). Diffusion imaging has shown that white matter microstructure is damaged in schizophrenia with reproducible patterns (Kochunov and Hong, 2014; Ohtani et al., 2014; Kochunov et al., 2022). The dMRI studies have indicated that white matter abnormalities are present throughout the whole process of schizophrenia, which is consistent with a developmental theory (Cetin-Karayumak et al., 2020).

Alzheimer’s disease is a neurodegenerative disease characterized by pathological changes in the gray and white matter. Cerebrovascular disease and AD pathology are commonly comorbid, significantly increasing the risk of developing dementia (Toledo et al., 2013; Arvanitakis et al., 2016; Azarpazhooh et al., 2018). White matter degeneration is closely related to cognitive and functional decline and may occur years before the onset of anticipated symptoms of AD (Ble et al., 2006; Lee et al., 2016; Zavaliangos-Petropulu et al., 2019). White matter degeneration is recognized as a prominent characteristic and potential biomarker of AD (Sachdev et al., 2013; Lee et al., 2016) and correlates with neuroimaging biomarkers of AD (Gaubert et al., 2021). Oligodendrocyte lineage cells dysfunction is a crucial mechanism of the white matter changes of AD and may be the potential target (Nasrabady et al., 2018; Lorenzini et al., 2020).

Multiple sclerosis is a chronic inflammatory, demyelinating disease with various clinical courses (Lassmann, 2018). Two types of inflammation may lead to demyelination in MS (Lassmann, 2018). Primary demyelination and diffuse neurodegeneration, which are two of the pathological features, involve the white and gray matter (Kutzelnigg et al., 2005; Haider et al., 2014; Lassmann, 2018). Remyelination of different extents helps repair demyelination (Patrikios et al., 2006; Patani et al., 2007). In fact, pre-existing unaffected oligodendrocytes rather than new oligodendrocytes perform remyelination (Yeung et al., 2019). However, most patients with MS cannot enhance oligodendrocyte production despite considerable potential (Yeung et al., 2019). Prompt and aggressive treatment is essential to protect oligodendrocytes and promote recovery (Giovannoni et al., 2016).

Microglia are resident myeloid cells with multiple functions and phenotypes (Lee et al., 2019). They have multiple functional subsets associated with the category or spatiotemporal state of the diseases and thus have numerous effects on white matter diseases (Lee et al., 2019). Oligodendrocyte lineage cells are affected by various subpopulations of microglia, both in the demyelination and remyelination phases (Li et al., 2017; Dai et al., 2020).

Oligodendrocytes, which have multiple functions, can produce myelin sheaths that can accelerate conduction velocity, making contributions to remyelination (Hamanaka et al., 2018; Kuhn et al., 2019). As oligodendrocyte progenitor cells (OPCs) can proliferate and differentiate into oligodendrocytes, OPCs are critical to oligodendrocytes regeneration (Clayton and Tesar, 2021). Oligodendrocyte lineage cells, which comprised oligodendrocytes and OPCs, can perform the reparative function with the help of the cells of the NVU (Levit et al., 2020). As oligodendrocyte lineage cells have multiple effects (Zhou et al., 2021), they are potential targets for the treatment of white matter diseases (Ohtomo et al., 2018).

### Emerging Trends of Publications on White Matter Hyperintensities

Burst detection indicates emerging trends in the field of study. Among the top 25 keywords with the strongest citation bursts, DL had the maximum burst strength, followed by ML, PVS, CNN, NVU, and NODDI.

Deep learning, which is a tool for ML and can analyze big data, is widely used in many areas of medical image analysis (Anwar et al., 2018). Currently, DL tractography segmentation method utilizing DL techniques performs well in segmenting white matter fibers (Poulin et al., 2019). The deep white matter analysis method leveraging DL can identify white matter tracts consistently and quickly (Zhang et al., 2020). DL can segment WMHs and other disease areas in CSVD (Duan et al., 2020; Shan et al., 2021). ML algorithms are employed to produce streamlines in the process of tractography; the path-based and local-model-free method can avoid the disadvantages of traditional tractography methods (Poulin et al., 2019). ML tractography will make significant contributions to white matter tractography; however, an outstanding framework for training and evaluating ML tractography methods is required (Poulin et al., 2019). CNNs are the most popular DL techniques used in medical image analysis (Anwar et al., 2018). CNN algorithms can segment WMHs and perform white matter tract segmentation efficiently with high accuracies (Li H. et al., 2018; Wasserthal et al., 2018; Schirmer et al., 2019; Li et al., 2020).

Perivascular spaces, which surround cerebral deep perforating arteries, are compartments containing cerebrospinal fluid. They can facilitate material exchange and waste clearance, and play an essential role in glymphatic function (Iliff et al., 2012). There were topological associations and volumetric associations between deep WMH lesions and PVSs, and PVSs may grow into WMHs; the underlying mechanism may be increased interstitial fluid and glymphatic dysfunction (Chen et al., 2019; Huang et al., 2021). PVS is a neuroimaging marker for CSVD (Chen et al., 2019).

Neurovascular unit includes neurons, astrocytes, endothelial cells, pericytes, vascular smooth muscle cells, and extracellular matrix components (Muoio et al., 2014). The NVU directs neurovascular coupling to match the metabolic demands (Girouard and Iadecola, 2006; Muoio et al., 2014). The blood brain barrier, the critical structure of the NVU, manages the diffusion of molecules between the blood and brain (Daneman and Prat, 2015). NVU dysfunction has adverse effects on central nervous system homeostasis and may be the underlying mechanism of WMHs (Cannistraro et al., 2019; Levit et al., 2020; Sabayan and Westendorp, 2021). Oligodendrocyte lineage cells supported by cells of the NVU make significant contributions to white matter repair (Hamanaka et al., 2018). Glial cells are expected to be therapeutic targets of WMHs (Levit et al., 2020).

Neurite orientation dispersion and density imaging is a dMRI approach with a multi-compartment model (Zhang et al., 2012). The neurite density index and orientation dispersion index, which are the essential parameters of NODDI, can evaluate neurite packing density and neurite angular variation and have been histologically validated (Zhang et al., 2012; Mollink et al., 2017; Schilling et al., 2018; Wang et al., 2019). NODDI is becoming popular, particularly in the field of white matter studies (Lynch et al., 2020; Beck et al., 2021; Lehmann et al., 2021).

### Strengths and Limitations

To our knowledge, this is the first study that quantitatively analyzed the publications on WMHs from 2012 to 2021 using the bibliometric analysis method. The intellectual structure and emerging trends were identified to provide directions for future studies on WMHs. However, this study has some limitations. Firstly, the publications were obtained only from the SCI-EXPANDED of WoS, and not from any other databases. Secondly, only publications in English were obtained, whereas publications in different languages were excluded. Thirdly, only original articles and reviews were retrieved, whereas other types of publications were not obtained. Last but not least, as the COVID-19 pandemic has had a major impact on scientific research and clinical practice, the trends of WMH study may have changed to some extent. Thus, the emerging trends identified in the current study may only be used as a reference.

## Conclusion

This study quantitatively analyzed the publications on WMHs in the past decade, using bibliometric analysis performed by Citespace and VOSviewer software. The study provided insights into the intellectual structure of WMH studies, and shed light on emerging trends. WMH studies mainly focused on the study of diseases with WMHs as the critical pathogenesis, dMRI research, and the mechanistic research of WMHs from the perspective of microglia and oligodendrocyte. The emerging trends centered on the application of new technologies such as ML in neuroimaging research. Studies on PVS and NVU were also identified as the emerging trends of WMH studies. This study can help researchers and clinicians obtain the hotspots and emerging trends of WMH studies and promote the collaboration among academics, institutions, and countries. Our findings can also provide direction for future basic and clinical studies of WMHs and promote the prevention and treatment of WMHs.

## Data Availability Statement

The raw data supporting the conclusions of this article will be made available by the authors, without undue reservation.

## Author Contributions

YS conceived and designed the study, performed the data analysis, and drafted the manuscript. ZZ, HT, and YS collected the data. SH supervised the study and revised the manuscript. All authors read and approved the submitted version.

## Conflict of Interest

The authors declare that the research was conducted in the absence of any commercial or financial relationships that could be construed as a potential conflict of interest.

## Publisher’s Note

All claims expressed in this article are solely those of the authors and do not necessarily represent those of their affiliated organizations, or those of the publisher, the editors and the reviewers. Any product that may be evaluated in this article, or claim that may be made by its manufacturer, is not guaranteed or endorsed by the publisher.

## Footnotes

1
http://bibliometric.com/
