# Supplementary figures and images for: Intellectual Structure and Emerging Trends of White Matter Hyperintensity Studies: A Bibliometric Analysis From 2012 to 2021
Source: Front Neurosci. 2022 Apr 11;16:866312. doi: 10.3389/fnins.2022.866312 (PMC9036105; doi:10.3389/fnins.2022.866312)

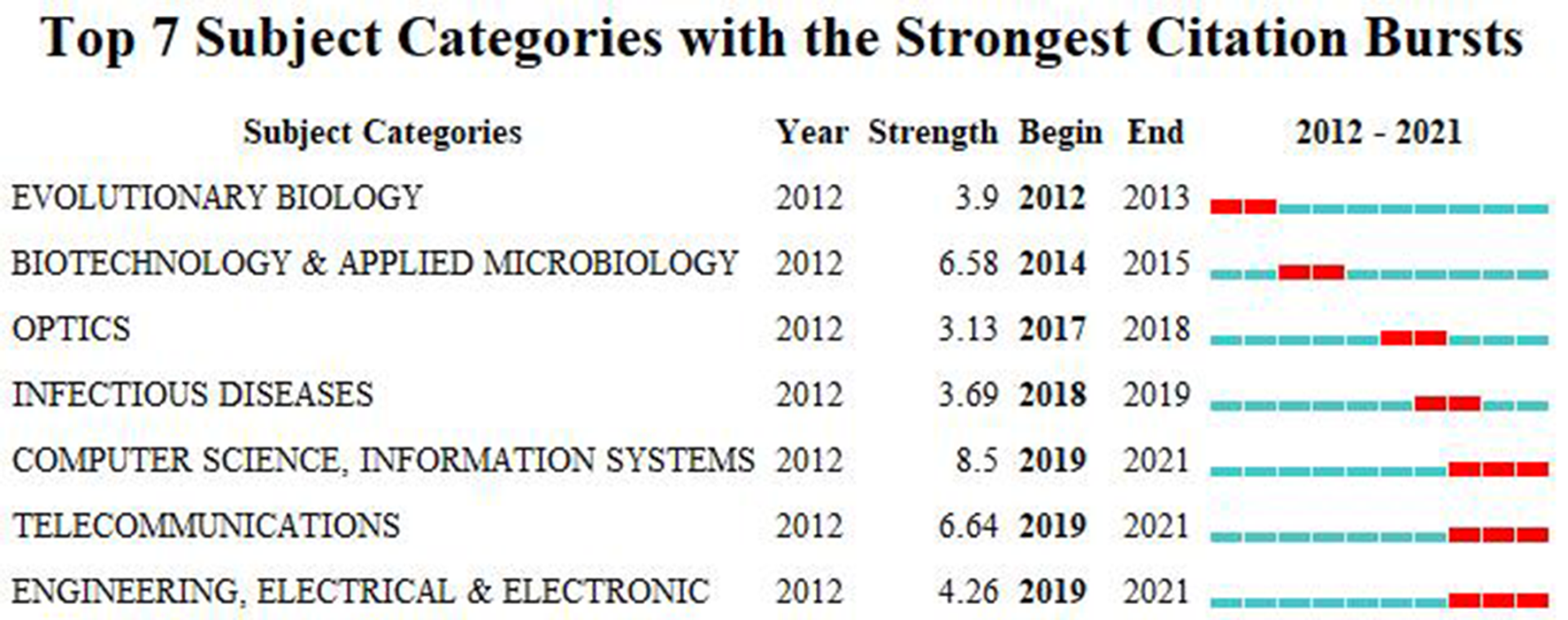

Supplement: Supplementary file 1 [file Image_1.tif]
